# High-throughput enrichment of temperature-sensitive argininosuccinate synthetase for two-stage citrulline production in *E. coli*

**DOI:** 10.1016/j.ymben.2020.03.004

**Published:** 2020-07

**Authors:** Thorben Schramm, Martin Lempp, Dominik Beuter, Silvia González Sierra, Timo Glatter, Hannes Link

**Affiliations:** Max Planck Institute for Terrestrial Microbiology, Karl-von-Frisch-Strasse 16, 35043, Marburg, Germany

**Keywords:** Temperature-sensitive enzymes, Citrulline overproduction, Single-cell growth, Flow cytometry, Two-stage bioprocess

## Abstract

Controlling metabolism of engineered microbes is important to modulate cell growth and production during a bioprocess. For example, external parameters such as light, chemical inducers, or temperature can act on metabolism of production strains by changing the abundance or activity of enzymes. Here, we created temperature-sensitive variants of an essential enzyme in arginine biosynthesis of *Escherichia coli* (argininosuccinate synthetase, ArgG) and used them to dynamically control citrulline overproduction and growth of *E. coli*. We show a method for high-throughput enrichment of temperature-sensitive ArgG variants with a fluorescent TIMER protein and flow cytometry. With 90 of the thus derived ArgG variants, we complemented an ArgG deletion strain showing that 90% of the strains exhibit temperature-sensitive growth and 69% of the strains are auxotrophic for arginine at 42 °C and prototrophic at 30 °C. The best temperature-sensitive ArgG variant enabled precise and tunable control of cell growth by temperature changes. Expressing this variant in a feedback-dysregulated *E. coli* strain allowed us to realize a two-stage bioprocess: a 33 °C growth-phase for biomass accumulation and a 39 °C stationary-phase for citrulline production. With this two-stage strategy, we produced 3 g/L citrulline during 45 h cultivation in a 1-L bioreactor. These results show that temperature-sensitive enzymes can be created *en masse* and that they may function as metabolic valves in engineered bacteria.

## Highlights

• Method to enrich temperature-sensitive enzymes *en masse*.
• Temperature-sensitive enzymes function as metabolic valve.
• Temperature controlled two-stage production of citrulline.

## Graphical abstract

**Figure:**
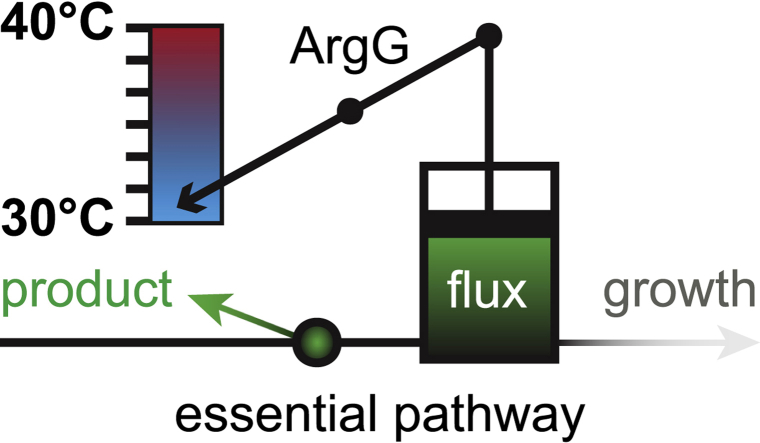

## 1 Introduction

The ability to switch overproduction strains between different physiological states enables bioprocesses with separate phases of growth and production (Brockman and Prather, 2015a; Burg et al., 2016; Klamt et al., 2018; Lalwani et al., 2018; Li et al., 2016). A common parameter to control growth and production is the concentration of nutrients in bioreactors, which affects physiology and metabolism of production strains (Mears et al., 2017; Michalowski et al., 2017; Tokuyama et al., 2019). Alternatively, it is also possible to control metabolism directly by engineering enzymes that act as metabolic valves. To this end, either the abundance or activity of the respective enzyme responds to external parameters, such as light (Zhao et al., 2018), chemicals/auto-inducers (Brockman and Prather, 2015b; Gupta et al., 2017; Soma et al., 2014), or temperature (Harder et al., 2018).

Temperature-responsive control of metabolism is economically advantageous since most bioreactors are standardly equipped with temperature regulation. Another advantage is that temperature dependent control is abundant in nature and occurs at DNA, RNA, and protein level (Klinkert and Narberhaus, 2009). As a consequence, many mutants of different organisms are known that show temperature-sensitivity (Li et al., 2011; Lovato et al., 2009; Saluja and Godson, 1995). In yeast, collecting known and creating new temperature-sensitive mutants led to large strain libraries, which can be applied to systematically investigate the function of essential genes (Ben-Aroya et al., 2008; Kofoed et al., 2015; Li et al., 2011).

Many temperature-responsive systems use transcription factors that show thermal sensitivity. For example, the temperature-sensitive transcription factor RheA of *Streptomyces albus* changes its activity reversibly upon temperature changes (Servant et al., 2000) and was used to control gene expression in chicken embryo cells (Weber, 2003). Temperature-sensitive variants of the transcription factor TetR were created to control expression of a restriction endonuclease in *E. coli*, which is a toxic product (Pearce et al., 2017). Another temperature-sensitive transcription factor is cI857 from *Escherichia* virus Lambda, which has a mutation (A66T) that is thought to introduce thermal instability (Nauta et al., 1997). This transcription factor was used in a biotechnological application to control the TCA cycle in *E. coli* (Harder et al., 2018). In this study, a temperature downshift from 37 °C to 28 °C repressed expression of the TCA cycle enzyme isocitrate dehydrogenase and redirected metabolic flux from biomass formation to production of itaconic acid. As an alternative to temperature-sensitive control of transcription, RNA thermometers control enzyme abundance at the level of translation in *E. coli* (Neupert et al., 2008; Sen et al., 2017) and *Thermus thermophilus* (Verdú et al., 2019).

Apart from temperature-dependent control of transcription and translation, temperature-sensitive enzymes allow a more direct control of metabolism. At the non-permissive temperature, the enzyme is inactive, which can arrest growth if the enzyme is essential or it redirects metabolic flux. A temperature-sensitive variant of the fatty acid biosynthesis enzyme FabI was used to produce malonyl-CoA derived 3-hydroxypropionic acid (Lynch et al., 2016), as well as temperature-sensitive variants of the enzymes FabI, FabB and FabD for fatty acid production (Lynch et al., 2015). These temperature-sensitive enzyme variants were obtained by nitrosoguanidine- or ethyl methane sulfonate-based random mutagenesis techniques (Broekman and Steenbakkers, 1973; Harder et al., 1974; Russell and Pittard, 1971). A temperature-sensitive variant of glyceraldehyde-3-phosphate dehydrogenase (GapA) was used to control glycolysis (Cho et al., 2012). GapA mutants were generated by error-prone PCR and used to complement a *gapA* deletion strain. This made it possible to identify temperature-sensitive GapA variants by inspecting colony growth at 30 °C and 37 °C.

Here, we show a method for high-throughput enrichment of essential temperature-sensitive enzyme variants with the single-cell growth rate reporter TIMER (Beuter et al., 2018; Claudi et al., 2014). As a case study, we used argininosuccinate synthetase (ArgG) in the arginine pathway and created ArgG variants by error-prone PCR. The fluorescent TIMER protein allowed us to enrich temperature-sensitive variants from the ArgG library, and we characterized 90 ArgG variants in more detail.

We then used one ArgG variant to dynamically control growth and production of citrulline in *E. coli*. Citrulline is the substrate of ArgG and an essential intermediate in the arginine biosynthesis pathway. Citrulline plays an important role in human metabolism (Curis et al., 2005) and is used as a dietary supplement. Approaches to produce citrulline include biocatalysis with purified enzymes or whole cell catalysis (Kakimoto et al., 1971; Song et al., 2015; Yamamoto et al., 1974), as well as fermentation processes with *Corynebacterium glutamicum* or *Bacillus subtilis* (Eberhardt et al., 2014; Hao et al., 2015; Ikeda et al., 2009; Okumura et al., 1966). An engineered strain of *C. glutamicum* produced the highest titers reported in the literature: 27 g/L citrulline at a final OD of ca. 70 (Ikeda et al., 2009). To our knowledge, citrulline has not been overproduced with engineered *E. coli*. Here, we produced 3 g/L citrulline in a 1-L bioreactor using a two-stage process strategy, which had a growth phase at 33 °C and a production phase at 39 °C.

## 2 Materials and methods

Chemicals were ordered from Merck KGaA or Carl Roth GmbH & Co. KG. MATLAB R2017b (MathWorks, Inc.) and BD FACSDiva 8.0.1 (BD Biosciences) were used for data analysis.

### 2.1 Construction of plasmids

Strains, plasmids, and oligonucleotides are listed in Suppl. Table 1. Q5 High-Fidelity DNA polymerase (New England BioLabs Inc. (NEB)) was used in PCRs. Plasmids were constructed using Gibson Assembly Master Mix (NEB). DNA fragments for Gibson assembly were purified after agarose gel-electrophoresis (NucleoSpin Gel and PCR Clean-up Kit, Macherey-Nagel GmbH & Co. KG). The DNA Clean & Concentrator Kit (Zymo Research Europe GmbH) was used for DNA clean-up after PCRs or Gibson assemblies. Plasmids were isolated from liquid cultures with the GeneJET Plasmid Miniprep Kit (Thermo Fisher Scientific Inc.). Oligonucleotides were obtained from Eurofins Genomics Germany GmbH. The wild-type *argG* gene from *E. coli* was expressed from a low-copy plasmid pTS036-*argG* with the pSC101 origin of replication (Suppl. Fig. 1) derived from the plasmid pUA66-rrnBp (Zaslaver et al., 2006). The pTS036-*argG* plasmid carries a chloramphenicol resistance *cmR*. The *argG* gene is under control of a *tetR* inducable promoter (pLetO-1) (Lutz and Bujard, 1997) and a strong RBS (Elowitz and Leibler, 2000). For overexpression of *argG* and *argG*-G9, pTS049 and pTS050 were constructed with the pCA24N backbone from the ASKA library (Kitagawa et al., 2005). ArgG and ArgG-G9 had an N-terminal His-tag.

### 2.2 Construction of strains

The Δ*argG* and Δ*argR* strains originated from the KEIO collection (Baba et al., 2006). The kanamycin resistance was removed with FLP-recombinase on the pCP20 plasmid (Datsenko and Wanner, 2000). Gene deletions were propagated from KEIO strains to other strains by P1 phage transduction (Thomason et al., 2007). The *argA*-H15Y mutation was introduced into the genome of the Δ*argG* Δ*argR* strain with a CRISPR-cas9 based method (Reisch and Prather, 2015; Sander et al., 2019a). All modifications of genomic DNA were verified by sequencing.

### 2.3 Cultivations

M9 minimal medium was used that contained 5 g/L glucose, 42.2 mM Na_2_HPO_4_, 22 mM KH_2_PO_4_, 11.3 mM (NH_4_)_2_SO_4_, 8.56 mM NaCl, 1 mM MgSO_4_ x 7 H_2_O, 100 μM CaCl_2_ x 2 H_2_O, 60 μM FeCl_3_, 7.6 μM CoCl_2_ x 6 H_2_O, 7.1 μM MnSO_4_ x 2 H_2_O, 7 μM CuCl_2_ x 2 H_2_O, and 6.3 μM ZnSO_4_ x 7 H_2_O. When required, 50 μg/mL kanamycin, 100 μg/mL carbenecillin, 30 μg/L chloramphenicol, 50 μg/mL spectinomycin, or 0.2 μM anhydrotetracycline (aTc) were added to the medium. LB and M9 minimal medium plates contained 1.5% agar. The conversion factor optical density (OD) to cell dry weight was 0.37 g_DW_ OD^-1^ L^-1^. The OD was measured at 600 nm.

#### 2.3.1 Growth characterization of 90 strain carrying ArgG variants

0.5 mL LB precultures were inoculated with 90 randomly picked strains carrying ArgG variants from agar plate and incubated in a 2 mL deep-well plate, sealed with gas permeable foil (Diversified Biotech, Inc.), at 37 °C for 6 h under shaking at 220 rpm. Glycerol stocks were prepared from the LB precultures. 150 μL M9 cultures were inoculated 1:75 with the LB precultures and incubated in a transparent 96-well plate (Greiner Bio-One GmbH) for 20 h at 30 °C in a Biotek Epoch plate reader (BioTek Instruments, Inc.). The M9 cultures were re-diluted 1:30 and incubated for another 20 h at 42 °C.

#### 2.3.2 Probing conditional arginine auxotrophy of nine strains carrying ArgG variants

0.5 mL LB precultures were started from glycerol stocks and incubated in a 2 mL-deep well plate, sealed with gas permeable foil, for 6 h at 37 °C under shaking at 220 rpm. 0.5 mL M9 precultures were inoculated 1:75 with LB precultures and incubated in a 2 mL-deep well plate at 30 °C for 16 h under shaking at 220 rpm. 150 μL M9 main cultures, supplemented with and without 1 mM arginine, were inoculated 1:75 with the M9 precultures and incubated in a transparent 96-well plate at 42 °C in a plate reader.

#### 2.3.3 Citrulline production with argG deletion strains

5 mL LB precultures were inoculated from glycerol stocks and incubated for 6 h at 37 °C in a rotary shaker. 5 mL M9 precultures, supplemented with 1 mM arginine, were inoculated 1:500 with the LB precultures and incubated overnight at 37 °C in a rotary shaker. M9 precultures were washed twice by centrifugation (4000 rpm, 37 °C, 5 min) with 5 mL M9 medium and resuspended in 5 mL M9 medium. 40 mL M9 main cultures supplemented with 100 μM arginine were inoculated with the washed M9 precultures to a start OD of 0.05 and incubated in 500 ml shake flasks at 37 °C under shaking at 220 rpm. After 3 h, the M9 main cultures were washed twice with M9 medium to remove the arginine from the medium. Subsequently, cells were resuspended in 30 mL of fresh M9 medium to an OD of 0.05, and incubated at 220 rpm and 37 °C.

#### 2.3.4 Growth and citrulline production screening of nine engineered strains carrying ArgG variants

5 mL LB precultures were inoculated from glycerol stocks and incubated for 6 h at 37 °C under shaking at 220 rpm. 5 mL M9 precultures were inoculated 1:100 with the LB precultures and incubated for 16 h at 30 °C under shaking of 220 rpm. 10 mL M9 main cultures were inoculated with the M9 precultures to a start OD of 0.05. The 10 mL M9 main cultures were split up to four parts, each 2 mL, and transferred to 5 mL-culture tubes that were incubated at different temperatures (30 °C, 34 °C, 37 °C, and 42 °C) for 7 h under shaking at 220 rpm.

#### 2.3.5 Growth characterization of strains carrying the ArgG variant G9 and the wild-type ArgG

5 mL LB precultures were inoculated from glycerol stocks and incubated for 6 h at 37 °C in a rotary shaker. 40 mL M9 precultures were inoculated 1:200 and incubated in 500 mL shake flasks for 16 h at 30 °C and 220 rpm of shaking. The growing cultures were washed twice with M9 medium by centrifugation, resuspended, and diluted to yield 45 mL M9 main cultures with a start OD of 0.05. The 45 mL M9 main cultures were split up to three parts, each 14 mL, and transferred to 100 mL shake flasks that were incubated at three different temperatures (30 °C/35 °C/39 °C or 33 °C/37 °C/42 °C) under shaking at 220 rpm.

#### 2.3.6 Two-stage bioreactor cultivation

5 mL LB precultures were inoculated from glycerol stocks and incubated for 6 h at 37 °C in a rotary shaker. 40 mL M9 precultures were inoculated 1:500 with the LB precultures and incubated in 500 mL shake flasks overnight at 33 °C under shaking at 220 rpm. The M9 precultures were diluted in 100 mL M9 to an OD of 0.15 and further cultivated in 1 L shake flasks at 33 °C for 8 h under shaking at 220 rpm. Subsequently, 45 mL of the M9 precultures were washed twice with M9 medium by centrifugation, resuspended, and diluted to yield 500 mL M9 main cultures with a start OD of 0.05, which were supplemented with 12.5 g/L glucose and 4 g/L NH_4_Cl and incubated in bioreactors (BioFlo/CelliGen 115 and BioFlo 120, Eppendorf AG). After 13 h of incubation at 33 °C, the temperature was switched stepwise to 39 °C (30 min 33 °C–36 °C, 10 min 36 °C–38 °C, 38 °C–39 °C). After 20 h, 25 mL of a sterile 300 g/L glucose 30 g/L NH_4_Cl solution was fed to the M9 main cultures.

#### 2.3.7 Cultivation for proteome analysis at different temperatures

5 mL LB precultures were inoculated from glycerol stocks and incubated for 6 h at 37 °C in a rotary shaker. 50 mL M9 precultures were inoculated 1:200 and incubated in 500 mL shake flasks overnight at 30 °C and 220 rpm. The cultures were washed twice with M9 medium by centrifugation, resuspended, and diluted to yield 70 mL M9 main cultures with a start OD of 0.2. The 70 mL M9 main cultures were divided into three volumes, each 20 mL, and transferred to 100 mL shake flasks that were incubated at three different temperatures (33 °C/39 °C/42 °C) under shaking at 220 rpm.

### 2.4 Enrichment of the temperature-sensitive ArgG variants

The *argG* gene was mutagenized with error-prone PCR according to the manufactures manual (Jena Bioscience GmbH, JBS Error-Prone Kit #PP-102). The template for the error-prone PCR was linear *argG* DNA (final concentration in the PCR: 0.1 ng/μL), which was amplified from the *E. coli* BW25113 genome and purified by agarose gel-eletrophoresis. The mutagenized *argG* DNA was cleaned-up and inserted into the plasmid pTS036 with Gibson assembly (NEBuilder HiFi DNA Assembly Master Mix (NEB)). After DNA clean-up, electrocompetent cells (MegaX DH10β cells) were transformed with pTS036-*argG*(mutant), and plated on two 150 mm petri-dishes with LB agar. After incubation overnight at 37 °C, colonies from two plates were collected in 20 mL LB medium and the plasmids were isolated, resulting in the final *argG* variant library pTS036-*argG*(mutant). *E. coli* Δ*argG* carrying the TIMER plasmid (pBR322_TIMER) was transformed with the pTS036-*argG*(mutant) library by electroporation. After plating the cells, colonies were collected from the plates with LB medium to prepare glycerol stocks. For the selective enrichment of temperature-sensitive variants, 25 mL M9 cultures were inoculated from glycerol stock, diluted at different ratios, and incubated for 36 h at 30 °C under shaking at 220 rpm. The culture with a final OD of 0.1 was re-diluted at different ratios and incubated for 6 h at 42 °C under shaking at 220 rpm. Then, slow growing cells were sorted with fluorescence-activated cell sorting (FACS) on a BD FACS Aria Fusion (BD Biosciences, NJ, USA). 561-nm lasers, 600 long pass and 632/22 bandpass filters were used to detect the red fraction of TIMER. 488-nm lasers, 500 long pass and a 520/30 band pass filters were used for green fluorescence. To identify cells in the forward/side scatter plot, 488-nm lasers were used. The sorted cells were plated on M9 agar medium (150 mm petri-dishes) at different dilutions and incubated at 30 °C. The growth of 90 randomly picked strains was investigated in microtiter plates (Methods 2.3.1).

### 2.5 Protein purification and enzymatic characterization of *E. coli* argininosuccinate synthetase ArgG

5 mL TB precultures were inoculated from glycerol stocks and incubated overnight at 37 °C in a rotary shaker. 200 mL TB main cultures in 1 L shake flasks were inoculated to a start OD of 0.04 with TB precultures and incubated at 37 °C under shaking at 220 rpm. At OD 0.6, 2 mL 0.1 M IPTG was added to the cultures. The cultures were incubated overnight at 16 °C under shaking at 220 rpm. Cells from 16 °C overnight culture were harvested by centrifugation (4000 rpm, 4 °C, 30 min). Following work was conducted at 4 °C. Cells were resuspended in 2.8 mL LEW (50 mM NaH_2_PO_4_ 300 mM NaCl, pH 8.0), and 100 μL protease inhibitor and DNase 1 was added to aliquots of 0.7 mL. Cells were lysed by sonication (3 × 1 min, with 30 s of cooling breaks). After centrifugation (30 min, 4 °C, 17.000 g), the supernatant was used for protein purification with Protino Ni-TED 1000 Packed Columns (Macherey-Nagel). Proteins were eluted in the first elution step. The protein concentration in the eluate was determined with the Pierce Microplate BCA Protein Assay Kit (Thermo Fisher, #23252). Stock solutions were prepared with protein concentrations of 0.2 mg mL^-1^. ArgG enzyme assays were conducted in 1.5 mL reaction tubes. 10 μL of enzyme stock solution was added to 80 μL reaction buffer that contained at final concentrations 10 mM ATP, 10 mM MgCl_2_, 10 mM L-aspartate, and 20 mM HEPES (pH 7). To start the reaction, 10 μL of L-citrulline was added (final concentration 10 mM). The formation of the reaction product, argininosuccinate, was measured with LC-MS/MS.

### 2.6 Citrulline, argininosuccinate, and glucose measurements

Citrulline was measured in the whole cultivation broth. Therefore, 5 μL cultivation broth was transferred to 495 μL 40:40:20 acetonitrile:methanol:H_2_O at -20 °C in 1.5 mL reaction tubes. Samples were centrifuged at -9 °C and 17.000 g for 20 min, and the supernatant was stored at -80 °C until analysis with LC-MS/MS. Citrulline concentrations were determined with an Agilent 1290 Infinity II UHPLC system coupled to an Agilent 6495 triple quadrupole mass spectrometer (Agilent Technologies, Inc.) (Guder et al., 2017). The LC column was an Acquity BEH Amide, 30 × 2.1 mm, 1.7 μm particle size, column (Waters Corporation). The mobile phases were (A) water with 10 mM ammonium formate and 0.1 vol.-% formic acid and (B) acetonitrile with 0.1 vol.-% formic acid. The flow rate was 0.4 mL min^-1^ and the gradients were: 0 min 90% B; 1.3 min 40% B; 1.5 min 40% B; 1.7 min 90% B; 2 min 90% B. Citrulline was quantified with a^15^N-internal standard. The ^14^N parent mass for citrulline was 176 and the product mass was 70, at a collision energy of 25 eV in positive mode (179 and 71 for ^15^N citrulline).

Samples of ArgG enzyme assays were prepared by transferring 10 μL of the enzyme assay reaction solution to 90 μL of 45:45:10 acetonitrile:methanol:water at -20 °C. Samples were stored at -80 °C until centrifugation (17.000 g, -9 °C). Argininosuccinate concentrations in the supernatants were determined by LC-MS/MS (Guder et al., 2017). The LC column was a HILICON iHILIC-Fusion(p), 50 × 2.1 mm, 5 μm particle size column. The mobile phases were (A) aqueous 10 mM ammonium carbonate and 0.2% ammonium hydroxide and (B) acetonitrile. The flow rate and gradients were the same as for citrulline quantification. Argininosuccinate was quantified with a^13^C-internal standard. The ^12^C parent mass for argininosuccinate was 291 and the product mass 70, at a collision energy of 45 eV in positive mode (301 and 74 for ^13^C argininosuccinate).

Supernatant samples for glucose measurements were prepared by centrifugation of 1 ml of the culture broth for 1 min at 17.000 g at room temperature. The supernatant was stored at -20 °C. The glucose concentration in the supernatant was determined with an assay kit (D-Glucose Assay Kit, GOPOD Format, Megazyme Inc).

### 2.7 Proteomics

Samples were subjected to tryptic digest as described in detail in the Supplementary Methods S1. 1 μg peptide was analyzed using liquid chromatography-mass spectrometry (LC-MS/MS). The LC-MS/MS analysis including label-free quantification was carried out as previously described in (Sander et al., 2019a) with minor modifications. In short, LC-MS/MS analysis of protein digests was performed on Q-Exactive Plus mass spectrometer connected to an electrospray ion source (Thermo Fisher Scientific). Peptide separation was carried out using Ultimate 3000 nanoLC-system (Thermo Fisher Scientific), equipped with packed in-house C18 resin column (Magic C18 AQ 2.4 μm, Dr. Maisch). The peptides were first loaded onto a C18 precolumn (preconcentration set-up) and then eluted in backflush mode with a gradient from 98% solvent A (0.15% formic acid) and 2% solvent B (99.85% acetonitrile, 0.15% formic acid) to 30% solvent B over 115 min. Label-free quantification was done using Progenesis QI software (Nonlinear Dynamics, v2.0), MS/MS search was performed in MASCOT (v2.5, Matrix Science) against the Uniprot *E. coli* protein database. The following search parameters were used: full tryptic search with two missed cleavage sites, 10 ppm MS1 and 0.02 Da fragment ion tolerance. Carbamidomethylation (C) as fixed, oxidation (M) and deamidation (N,Q) as variable modification. Progenesis outputs were further processed with SafeQuant (Glatter et al., 2012).

## 3 Results

### 3.1 Enrichment of temperature-sensitive ArgG variants with a TIMER protein

**Fig. 1:**
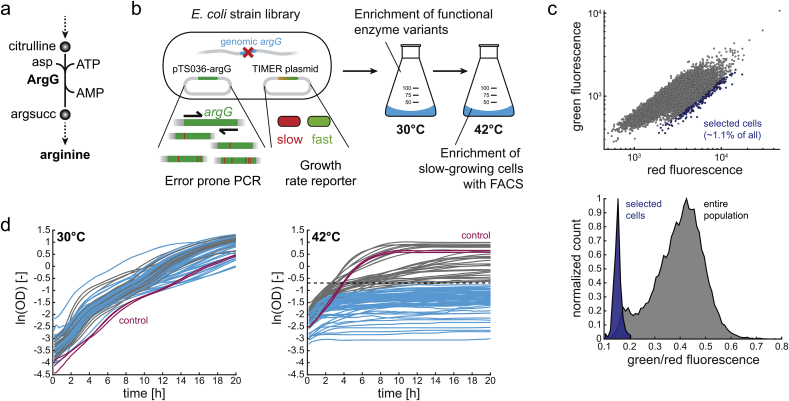
High-throughput enrichment of temperature-sensitive ArgG variants. (a) ArgG catalyzes the seventh reaction in the arginine biosynthesis pathway and converts citrulline into argininosuccinate (argsucc). asp, aspartate. (b) A plasmid library with mutagenized *argG* was generated with error-prone-PCR and used to complement an *argG* knockout strain of *E. coli* BW25113. The strain also carried a plasmid with the single-cell growth rate reporter TIMER. The pooled strain library was first incubated at 30 °C to enrich ArgG variants that are catalytically active and support growth. Subsequently, the culture was shifted to 42 °C to select non-growing cells with FACS based on the green/red signal of the TIMER protein. (c) Red and green fluorescence (top) of cells after 6 h culturing at 42 °C. FACS was used to isolate the fraction with a low green/red ratio (shown in blue). Histogram (bottom) showing the distribution of cells according to the green to red ratio. (d) Growth at 30 °C and 42 °C of 90 single strains isolated from the fraction with a low green/red ratio (blue fraction in Fig. 1c). Strains that did not reach an OD of 0.5 at 42 °C are shown in blue. A control strain expressing wild-type ArgG is shown in red. (For interpretation of the references to colour in this figure legend, the reader is referred to the Web version of this article.)

Argininosuccinate synthetase (ArgG) catalyzes the seventh reaction in the arginine biosynthesis pathway of *E. coli*, and converts citrulline into argininosuccinate (Fig. 1a). To construct temperature-sensitive variants of ArgG, we used error-prone PCR for in vitro mutagenesis of the *argG* gene (Fig. 1b). The resulting library of ArgG variants was then used to complement an *argG* deletion strain (Δ*argG*) (Baba et al., 2006). The Δ*argG* strain is auxotrophic for arginine and does not grow on a minimal glucose medium without arginine (Suppl. Fig. 2). Therefore, only cells with catalytically active ArgG variants would grow on glucose minimal medium, and we enriched these variants by culturing the library for 36 h at 30 °C in shaking flasks. Then, we shifted the culture to 42 °C and expected a growth-arrest of cells expressing temperature-sensitive ArgG variants (variants that are inactivated at 42 °C). The challenge was to isolate the fraction of growth-arrested cells from the library, and for this purpose, we used the single-cell growth rate reporter TIMER. The TIMER protein is a dsRed variant, which indicates slow- and non-growing cells by a low ratio of green/red fluorescence (Beuter et al., 2018; Claudi et al., 2014). After switching the ArgG library from 30 °C to 42 °C, we waited 6 h to allow maturation of the TIMER protein and then isolated 81.000 cells with the lowest green/red ratio with FACS (Fig. 1c). The entire ArgG library had a wide-range of green/red ratios with a ratio median of 0.44, while the sorted fraction had a narrow green/red ratio with a median of 0.14. This indicates that the majority of cells was growing at 42 °C and did not express temperature-sensitive ArgG variants.

To test if we enriched temperature-sensitive ArgG variants, we randomly selected 90 isolates from the sorted cells with low green/red ratios. The 90 isolates were then cultured in microtiter plates at both 30 °C and 42 °C. At 30 °C, all isolates grew similar to a control strain that expressed the wild-type ArgG, thus indicating that all ArgG variants are catalytically active at 30 °C (Fig. 1d and Suppl. Fig. 3). At 42 °C in contrast, the majority of strains (90%) grew worse than the control strain, and 62 out of the 90 strains did not reach a final OD of 0.5. Thus, the single cell growth rate reporter successfully enriched temperature-sensitive ArgG variants. Moreover, the large variation of growth characteristics at 42 °C suggests that the enzymes respond differently to a temperature increase.

### 3.2 Characterization of nine temperature-sensitive ArgG variants

**Fig. 2:**
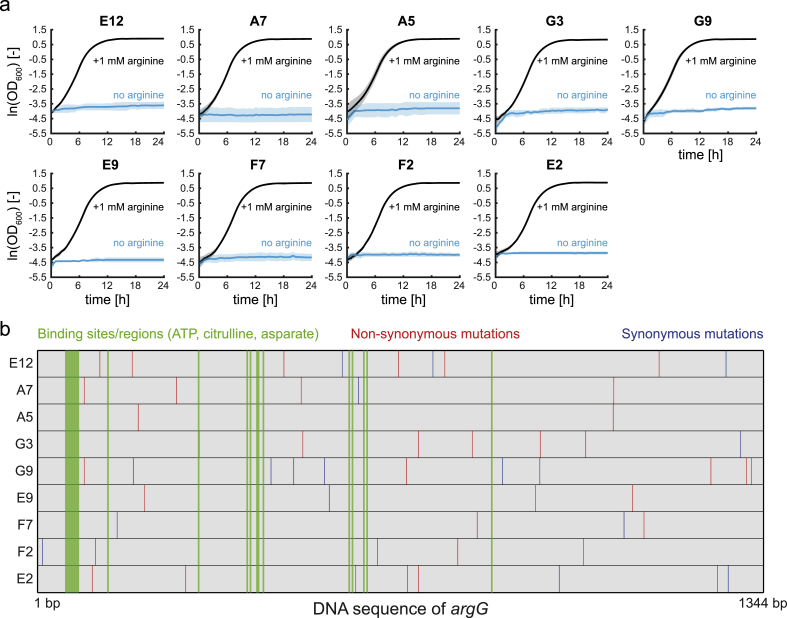
Temperature-dependent arginine auxotrophy and mutations of nine ArgG variants. (a) Growth of nine strains with ArgG variants at 42 °C with supplementation of arginine (black lines) and without (blue lines). Lines show means and shades the standard deviation of n = 3 plate reader cultures. (b) Mutations of the nine ArgG variants. Binding sites of ATP, citrulline, and aspartate are shown in green. Non-synonymous mutations are red, synonymous are blue. (For interpretation of the references to colour in this figure legend, the reader is referred to the Web version of this article.)

We selected nine ArgG variants that did not support growth at 42 °C but achieved the highest growth rates at 30 °C (Suppl. Fig. 4). First, we confirmed that the nine strains are indeed auxotrophic for arginine at 42 °C by culturing them with and without supplementation of arginine (Fig. 2a). Addition of arginine could fully restore growth at 42 °C, with maximum growth rates of 0.70 ± 0.02 h^-1^. Consequently, the temperature-sensitivity of all nine strains results from an auxotrophy for arginine.

Sequencing of the nine ArgG variants revealed that each variant had between two and eleven mutations (Fig. 2b and Suppl. Table 2). 69% of all mutations were non-synonymous and changed the amino acid sequence. All ArgG variants were unique, and only a few ArgG variants had amino acid exchanges at the same position (G9 and A7 at position 29, E2 and G3 at position 235, and A5 and A7 at position 356). The E235D mutation occurred twice in G3 and E2, and mutant A7 had a point mutation in the ribosomal binding site of *argG*. The number of synonymous mutations per gene varied between zero (variant A5) and six (variant G9). Because codon usage has an impact on the translational efficiency (Plotkin and Kudla, 2011), we expect that these mutations could play a role in tuning expression levels of ArgG. However, it seems unlikely that ArgG expression levels play a role in temperature-sensitivity, because this would also affect growth at 30 °C. Mapping the mutations to the DNA sequence of *argG* revealed that only one out of 55 mutations (F100Y of G3) occurred at a known binding site for citrulline, ATP, or aspartate (Lemke and Howell, 2002, 2001) indicating conservation of those critical residues.

To test if these mutations have the potential to reduce protein stability we obtained ΔΔ*G* values with FoldX (Guerois et al., 2002; Schymkowitz et al., 2005). ΔΔ*G* are changes in Gibbs energy (Δ*G*) relative to the wild-type ArgG sequence. The average ΔΔ*G* for the nine mutants was 6.5 kcal mol^-1^ (Suppl. Table 2), which indicated strong destabilizing effects. As a reference, previous studies showed that the average ΔΔ*G* for single amino acid substitution in 22 proteins was 1.3 kcal mol^-1^, and only 15% of these mutations had a ΔΔ*G* >3 kcal mol^-1^ (Tokuriki et al., 2008).

Next, we used the nine ArgG variants to dynamically control the arginine pathway and to overproduce the substrate of ArgG, citrulline.

### 3.3 Citrulline overproduction in feedback-dysregulated *E. coli*

**Fig. 3:**
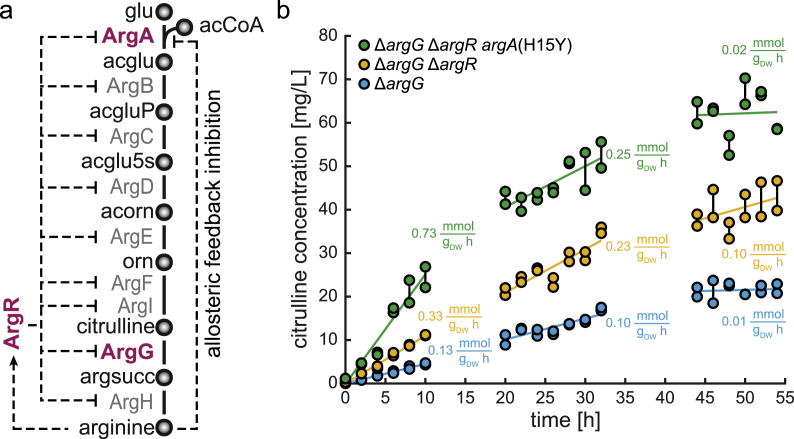
Overproduction of citrulline in an *argG* deletion strain. (a) Arginine biosynthesis in *E. coli* is feedback regulated by arginine at the level of transcription (ArgR) and allosteric control of the first enzyme (ArgA). The transcription factor ArgR regulates the expression of all genes in the pathway. The activity of the protein ArgA, which catalyzes the first reaction in the pathway, is directly regulated by allosteric interaction with arginine. Engineering targets are shown in red: deletion of *argG*, deletion of *argR*, H15Y mutation removes allosteric inhibition of ArgA. glu, L-glutamate; acglu, N-acetyl-L-glutamate; acgluP, N-acetylglutamyl-phosphate; acglu5s, N-acetyl-L-glutamate 5-semialdehyde; acorn, N-acetyl-L-ornithine; orn, L-ornithine; argsucc, L-arginino-succinate; acCoA, acetyl-coenzyme-A; (b) Citrulline concentration in the whole cultivation broth of three *argG* deletion strains after removing arginine from the cultivation medium. Blue: a strain with only *argG* deletion, Orange: a strain with *argG* deletion and additional deletion of the transcriptional repressor *argR*. Green: a strain with deletion of *argG*, *argR* and a point mutation (H15Y) in *argA* that removes inhibition of ArgA by arginine. Specific citrulline production rates were calculated by regression analysis in the three time intervals. (For interpretation of the references to colour in this figure legend, the reader is referred to the Web version of this article.)

We expected that inactivation of ArgG blocks the arginine pathway and that this would lead to an accumulation of the substrate of ArgG, citrulline (Fig. 3a). To test the consequences of blocking arginine biosynthesis at the reaction catalyzed by ArgG (argininosuccicante synthetase), we used an *argG* deletion strain (Δ*argG*). First, the Δ*argG* strain was grown in medium with externally supplemented arginine, which suppresses the physiological consequences of the *argG* deletion. Then, we washed and diluted the cells in medium without arginine so that cells would have to switch to *de novo* arginine biosynthesis. After dilution, we measured the OD and citrulline concentration in the whole cultivation broth for 54 h (Fig. 3b). As expected, cells did not grow after removing arginine from the medium, and the OD remained constant at 0.05 (Suppl. Fig. 2). Citrulline, in contrast, increased to a final concentration of 20 mg/L, showing that blocking ArgG results in accumulation of citrulline.

To improve citrulline production, we dysregulated the arginine pathway, which is feedback regulated at the transcriptional and allosteric level by arginine (Fig. 3a). Therefore, we deleted the transcriptional repressor of the arginine pathway (ArgR) and removed allosteric feedback inhibition by a point mutation in the first enzyme in the pathway (H15Y mutation of ArgA) (Rajagopal et al., 1998). Deletion of ArgR resulted in a 2-fold higher citrulline concentration at the end of the experiment (40 mg/L) (Fig. 3b). A doubly dysregulated strain (Δ*argG* Δ*argR argA*^H15Y^) achieved 3-fold higher citrulline concentrations (60 mg/L). The specific citrulline production rates of the three strains decreased over time. During the first 10 h, the doubly dysregulated strain had a biomass-specific citrulline production rate of 0.73 mmol g_DW_^-1^ h^-1^, which decreased to 0.25 mmol g_DW_^-1^ h^-1^ between 20 and 32 h. Citrulline production stopped between 44 and 54 h.

These results show that blocking arginine biosynthesis at the ArgG reaction leads to overproduction of citrulline, and that complete dysregulation of the arginine pathway enhances production. Next, we combined the doubly dysregulated strain (Δ*argG* Δ*argR argA*^H15Y^) with temperature-sensitive ArgG to control citrulline production dynamically.

### 3.4 Growth and citrulline production screening of the temperature-sensitive ArgG variants

**Fig. 4:**
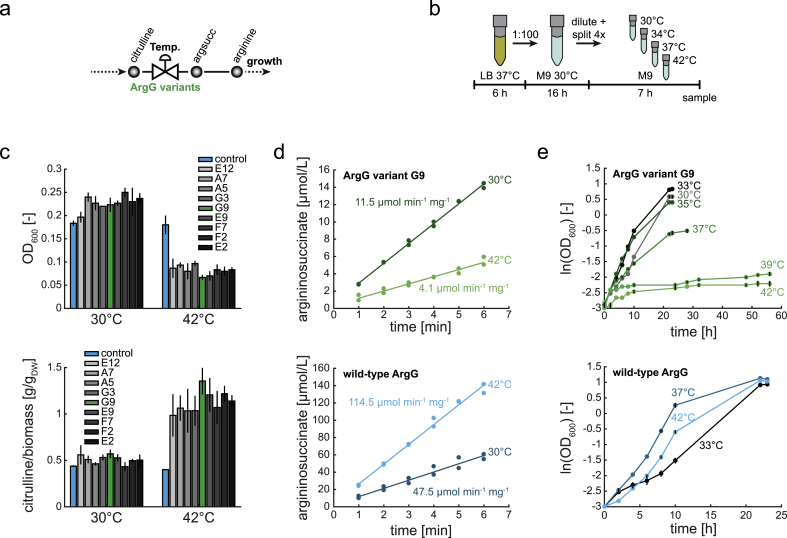
Growth and citrulline production of the doubly dysregulated citrulline producer in combination with the nine ArgG variants. (a) Schematic of a temperature-sensitive metabolic valve at ArgG. (b) Schematic of the experimental setup to screen citrulline production and growth of nine ArgG variants at different temperatures. (c) The nine ArgG variants were expressed in the doubly dysregulated citrulline producer (Δ*argG* Δ*argR argA*^H15Y^). Shown is the OD (top) after 7 h cultivation in minimal medium at 30 °C and 42 °C. All cultures started at an OD of 0.05. Error bars show the standard deviation of n = 3 cultures. Biomass specific citrulline concentration (bottom) after 7 h cultivation in minimal medium at 30 °C and 42 °C. Error bars show the standard deviation of n = 3 cultures. (d) *In vitro* enzymatic assays with purified ArgG variant G9 (top) and wild-type ArgG (bottom) at different temperatures (n = 2 enzyme assays, proteins purified 2 times). Shown is the formation of the reaction product (argininosuccinate) after starting the reaction at t = 0 min. Specific enzyme activities were calculated with linear regression. (e) Growth of the doubly dysregulated citrulline producer (Δ*argG* Δ*argR argA*^H15Y^) expressing the ArgG variant G9 (top), and wild-type ArgG (bottom). Colors indicate different temperatures. Dots are means, and error bars show the difference between n = 2 cultures. (For interpretation of the references to colour in this figure legend, the reader is referred to the Web version of this article.)

To control growth and overproduction of citrulline dynamically, we expressed the nine temperature-sensitive ArgG variants in the doubly dysregulated citrulline producer Δ*argG* Δ*argR argA*^H15Y^. As a control, we also expressed the wild-type ArgG enzyme in the same strain. We expected that temperature-sensitive ArgG functions as a metabolic valve that controls arginine biosynthesis flux in a temperature-dependent manner (Fig. 4a). At low temperatures, ArgG is active and sustains biosynthetic flux into arginine and growth. At high temperatures, ArgG is inactive and blocks the arginine pathway, resulting in overproduction of citrulline and a growth-arrest.

To test if the nine ArgG variants achieve this metabolic control in the citrulline producer, we cultured the nine strains and the control (Fig. 4b). M9 precultures (30 °C) were used to inoculate 4 main cultures at OD 0.05, which were incubated at different temperatures (30 °C/34 °C/37 °C/42 °C). OD and citrulline were measured after 7 h. At 30 °C, all strains with a temperature-sensitive ArgG variant grew to a similar OD, which was even higher than the OD of the control strain (Fig. 4c). Citrulline production at 30 °C was similar in all strains, including the control (Fig. 4c). The basal production of citrulline indicates that ArgG is limiting the flux in the arginine pathway already at 30 °C. This bottleneck at ArgG is probably caused by high abundances of the other enzymes in the arginine pathway due to the ArgR deletion (Sander et al., 2019b).

When we cultured the strains at 42 °C, the OD of all temperature-sensitive variants increased just slightly (from 0.05 to an average of 0.08). Citrulline production of the variants increased on average 2.2-fold at 42 °C when compared to 30 °C. In contrast, the control strain reached almost the same OD and citrulline levels at 42 °C and 30 °C. This shows that all ArgG variants enable temperature-dependent control of both growth and citrulline production.

Notably, at intermediate temperatures (34 °C and 37 °C), the OD and citrulline levels differed across the nine ArgG variants (Suppl. Fig. 5), suggesting that the variants have different temperature dependencies.

The ArgG variant G9 achieved the highest citrulline levels, and we tested temperature-sensitivity of this variant with in vitro enzyme assays. The results show that ArgG-G9 is indeed temperature-sensitive: the specific enzyme activity was 11.5 μmol min^-1^ mg^-1^ at 30 °C and 4.1 μmol min^-1^ mg^-1^ at 42 °C (Fig. 4d). In contrast, the activities of wild-type ArgG were 47.5 μmol min^-1^ mg^-1^ at 30 °C and 114.5 μmol min^-1^ mg^-1^ at 42 °C. The incubation time at 42 °C did not affect activity of the enzymes, because activities did not change when incubating the enzymes for 10 min or 60 min at 42 °C (wild type: 112.0 μmol min^-1^ mg^-1^, variant G9: 4.0 μmol min^-1^ mg^-1^) (Suppl. Fig. 6). This data indicated that temperature-sensitivity of the ArgG-G9 variant occurred at faster time-scales.

Cultivating the doubly dysregulated strain with ArgG variant G9 at different temperatures (30, 33, 35, 37, 39, 42 °C) revealed that the best growth occurred at 33 °C and that 39 °C was sufficient to stop growth (Fig. 4e). Between 33 °C and 39 °C, growth decreased gradually, showing tunable growth control of this ArgG variant by temperature. The control strain, which expressed the wild-type ArgG, grew at all temperatures and best at 37 °C (Fig. 4e).

Strains with ArgG-G9 grew faster than strains with wild-type ArgG at low temperatures (Fig. 4e and Suppl. Fig. 4) although the specific enzyme activity of ArgG-G9 was lower compared to the wild-type ArgG (Fig. 4d). A potential explanation is that the mutations (also synonymous) tuned and increased expression levels by codon usage or mRNA stability resulting in faster growth.

**Fig. 5:**
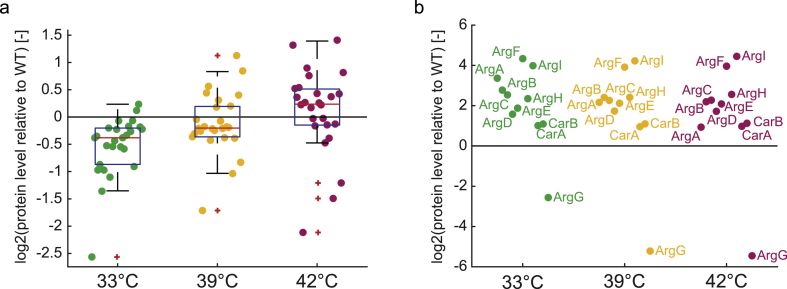
Proteome data of the doubly dysregulated citrulline producer (ΔargG ΔargR argA^H15Y^) expressing the ArgG variant G9 at different temperatures (33 °C, 39 °C, 42 °C). Data is normalized to the proteome of exponentially growing wild-type cells at 37 °C. Dots are the mean of independent replicates (n = 3). (a) Relative abundance of heat-shock proteins: IbpB, IbpA, DnaJ, GroS, DnaK, FxsA, GroL, ClpB, HtpX, HtpG, GrpE, Lon, YcjF, PrlC, HslV, MutM, HslU, YbbN, YbeZ, RpoD, YbeD, YcjX, LdhA, ClpP, ClpX, HslJ. Red lines indicate the medians. Boxes indicate the 25th and 75th percentiles. (b) Relative abundance of enzymes in the arginine biosynthesis pathway as well as CarA and CarB. (For interpretation of the references to colour in this figure legend, the reader is referred to the Web version of this article.)

Heat stress and the accompanying metabolic burden could potentially pose a major challenge in temperature-controlled bioprocesses. Therefore, we measured the proteome of our strains at different temperatures and investigated the abundance of heat-shock related proteins (Fig. 5a). Heat-shock related proteins were stronger expressed at 42 °C than at 39 °C, which suggested that switching to 39 °C was less burdensome than switching to 42 °C.

We then inspected the abundance of the proteins in the arginine pathway. All arginine enzymes increased in the doubly dysregulated strain (average 6.9-fold), which was expected due to deletion of ArgR (Fig. 5b). The only exception was ArgG-G9, because this enzyme was expressed from a plasmid. ArgG-G9 levels were 5.9-fold below wild-type levels during exponential growth at 33 °C. At 39 °C and 42 °C, ArgG-G9 levels decreased further, 6.3- and 7.4-fold, respectively. The lower abundance of ArgG-G9 at high temperatures indicated degradation of either the enzyme or the mRNA. Thus, it seems that high temperature affects ArgG-G9 two-fold: it reduces stability and abundance of the protein.

### 3.5 A temperature-controlled two-stage process for overproduction of citrulline

**Fig. 6:**
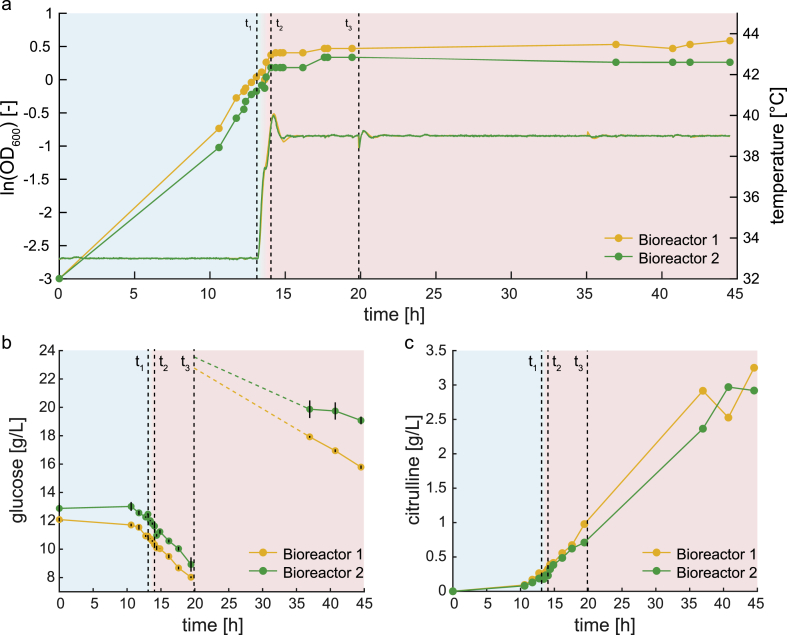
Two-stage production of citrulline with the ArgG variant G9. (a) The doubly dysregulated citrulline producer (Δ*argG* Δ*argR argA*^H15Y^) expressing the ArgG variant G9 was cultivated in two independent 1-L bioreactors. OD and the temperature are shown for bioreactor 1 (orange) and bioreactor 2 (green). t_1_ and t_2_ indicate the time window when temperature was increased from 33 °C to 39 °C. t_3_ indicates the time when glucose and ammonium was fed. blue area: growth phase, red area: production phase. (b) Glucose concentration in the supernatant of the two bioreactors. Dots are the mean, and error bars are the standard deviation of n = 4 analytical replicates per bioreactor. (c) Citrulline concentration in the whole cultivation broth of the two bioreactors. (For interpretation of the references to colour in this figure legend, the reader is referred to the Web version of this article.)

We used the doubly dysregulated strain (Δ*argG* Δ*argR argA*^H15Y^) with the temperature-sensitive ArgG variant G9 to produce citrulline in a 1 L-bioreactor. This strain grew best at 33 °C and growth stopped already at 39 °C. Therefore, we used these temperatures to separate a growth phase (33 °C) and a production phase (39 °C). We cultivated the strain in two independent bioreactors for 14 h at 33 °C in minimal medium until the biomass reached an OD of approximately 1. During this time, the cells grew exponentially with growth rates of 0.31 h^-1^ and 0.35 h^-1^. Then, we slowly increased the temperature to 39 °C over a time of 1 h. The slow temperature increase was necessary to avoid a temperature overshoot that may cause a heat shock. Growth stopped when both bioreactors reached 39 °C, and cells entered a stationary phase (Fig. 6a).

Throughout the experiment, we measured glucose and citrulline concentrations to quantify specific uptake and production rates. The glucose concentrations in the supernatant decreased continuously (Fig. 6b), and during the first 5 h into the stationary phase the specific glucose uptake rates were 4.2 mmol g_DW_^-1^ h^-1^ and 5.3 mmol g_DW_^-1^ h^-1^. These rates correspond to 45% of the glucose uptake rate of glucose-fed wild-type *E. coli* during exponential growth at 37 °C (Long et al., 2018). Thus, despite the growth arrest, cells remained a relatively high metabolic activity, which is about 45% of exponentially growing cells.

Apart from arresting growth, the temperature shift from 33 °C to 39 °C induced production of citrulline. During the 30 h stationary phase, specific citrulline production was constant at 0.92 mmol g_DW_^-1^ h^-^^1^ (bioreactor 1) and 1.10 mmol g_DW_^-1^ h^-1^ (bioreactor 2). These rates matched productions rates estimated from the initial screening (Fig. 4c) and were 38% higher than for the doubly dysregulated strain with deletion of ArgG (Fig. 3b). The final concentrations of citrulline in the two bioreactors were 3.3 g/L and 2.9 g/L (Fig. 6c), and the final biomass-specific citrulline yields were 4.88 g/g_DW_ and 6.07 g/g_DW_. Titers, yields, and production rates of all experiments are listed in Suppl. Table 3.

## 4 Discussion

We presented a method to enrich a large number of temperature-sensitive variants of an essential enzyme. The single-cell growth rate reporter TIMER was key to enrich thousands of potentially temperature-sensitive variants because it allowed us to isolate a small fraction of slow- or non-growing cells (1.1%) from a population of mainly growing cells. The ability to create temperature-sensitive enzymes *en masse* opens up opportunities to comprehensively map mutations that confer thermal sensitivity, for example by deep sequencing of all enzyme variants (Bassalo et al., 2018; Garst et al., 2017). Such approaches would advance our understanding about principles that underlie thermal sensitivity of enzymes (Leuenberger et al., 2017) and enable the prediction of temperature effects based on protein sequences (Chakshusmathi et al., 2004).

Apart from mapping mutations that confer thermal sensitivity, enriching a large number of temperature sensitive enzymes facilitates the identification of variants that are optimal for a particular application. For example, it could be possible to find enzymes that are partially inactive at a given temperature or enzymes that switch quickly forth and back upon temperature changes. Such gradual and reversible control of an enzyme by temperature would be an alternative to knockdown methods like CRISPR interference (Larson et al., 2013; Li et al., 2016; Qi et al., 2013), which requires expression of additional protein and RNA components in the host.

Our case study was the arginine biosynthesis enzyme argininosuccinate synthetase (ArgG), and we applied it for the overproduction of citrulline. Our data suggested that the ArgG variants have indeed different temperature-dependencies and that the temperature affects the ArgG catalyzed reaction, and consequently arginine biosynthesis, in a gradual and tunable way. This precise control of metabolic reactions and pathways renders temperature-sensitive enzymes an effective tool to implement metabolic valves in overproduction strains.

Recent computational studies identified targets that are particularly suited as metabolic valves in two-stage bioprocesses (Venayak et al., 2018), and the methods presented in this study can help implementing them in production strains. So far, the method is limited to valves that are essential proteins because selection of temperature-sensitive variants is dependent on growth. A solution to this problem is modifying strains such that non-essential targets become essential, for example by deletion of isoenzymes.

Creating many protein variants with different temperature-characteristics is especially important because computational analysis suggested that multiple metabolic valves are necessary to achieve optimal dynamic metabolic control for certain products (Burg et al., 2016; Venayak et al., 2018). A practical problem in implementing multiple metabolic valves is probably to coordinate their switching behavior with a single input signal like temperature. To this end, a large panel of temperature-sensitive enzymes, each with different temperature dependencies, will help addressing this problem and finding the optimal combination of valves.

We showed that temperature-sensitive ArgG functions as a metabolic valve and that it switches the arginine pathway between synthesis of the amino acid end-product (arginine) and synthesis of the intermediate (citrulline). The ArgG deletion strain revealed the consequences of completely closing the metabolic valve: a growth arrest and production of citrulline. Removing allosteric feedback inhibition and transcriptional regulation in the arginine pathway increased citrulline production about 3-fold indicating that even in the absence of arginine the pathway is feedback inhibited. This basal repression results either from the remaining arginine levels (e.g. from protein degradation) or inhibitory effects of other metabolites. For example, lysine has been shown to be an additional activator of the arginine repressor ArgR (Lempp et al., 2019). Temperature-sensitive ArgG allowed us to establish a two-stage bioprocess, in which the metabolic valve was open at 33 °C and closed at 39 °C. In the future, it will be important to clarify if partially closing the metabolic valve results in better overproduction and avoids premature decreases of production rates (here after 44 h in the ArgG deletion strain). Another option to maintain a high productivity might be switching continuously between different temperatures. Such an approach requires temperature-sensitive enzymes that switch reversibly between an active and inactive state.

Temperature gradients and fluctuations in large-scale fermentations might be problematic if they cause an unintended growth arrest during the growth phase (e.g. if zones with 42 °C exist during the 33 °C growth phase). Therefore, future studies could examine the function of temperature-sensitive enzymes under fluctuating temperatures. Economically feasible production of citrulline at an industrial-scale would require higher titers of citrulline, which can be realized by prolonging both growth and production phases. Further improvements can be achieved by genomic integration of temperature-sensitive variants, expression of exporters, and elimination of competing pathways (e.g. the putrescine pathway).

In conclusion, temperature-sensitive enzymes are a promising tool for metabolic engineering that enable dynamic control of metabolism. In case of temperature-sensitive ArgG, the biggest advantage is the ability to gradually switch the arginine biosynthetic pathway on and off. Thus, together with thermo-sensitive transcription or translation, temperature-sensitive enzymes open up novel applications and process strategies in industrial biotechnology.

## Funding

This work was supported by the ERC starting grant 715650.

## CRediT authorship contribution statement

**Thorben Schramm:** Conceptualization, Investigation, Visualization, Project administration, Writing - original draft, Writing - review & editing, Formal analysis. **Martin Lempp:** Investigation. **Dominik Beuter:** Investigation. **Silvia González Sierra:** Investigation, Resources. **Timo Glatter:** Investigation, Resources, Formal analysis. **Hannes Link:** Conceptualization, Supervision, Project administration, Visualization, Writing - original draft, Writing - review & editing, Formal analysis, Funding acquisition.

## Declaration of competing interest

None.
