# Supplementary material for: High-throughput enrichment of temperature-sensitive argininosuccinate synthetase for two-stage citrulline production in E. coli
Source: Metab Eng. 2020 Jul;60:14–24. doi: 10.1016/j.ymben.2020.03.004 (PMC7225747; doi:10.1016/j.ymben.2020.03.004)
Supplement: Multimedia component 1 [file mmc1.docx]

**SUPPLEMENTARY INFORMATION**

**High-throughput enrichment of temperature-sensitive argininosuccinate synthetase for two-stage citrulline production in *E. coli***

Thorben Schramm, Martin Lempp, Dominik Beuter, Silvia González Sierra, Timo Glatter, Hannes Link*

Max Planck Institute for Terrestrial Microbiology, Karl-von-Frisch-Strasse 16, 35043 Marburg, Germany

*Corresponding author.

E-mail address: hannes.link@synmikro.mpi-marburg.mpg.de (H. Link).

**Supplementary Figures**


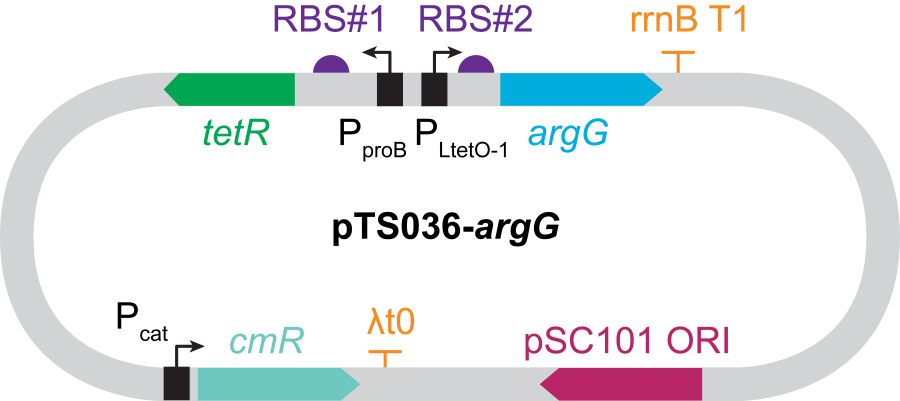


Suppl. Fig. 1. Plasmid map of pTS036-*argG*.

RBS#1 and P_proB_=ribosomal binding site and promoter of *proB* (Davis et al., 2011). RBS#2=strong ribosomal binding site (Elowitz and Leibler, 2000). P_LtetO-1_=*tetR* inducable promoter (Lutz and Bujard, 1997). Backbone derives from pUA66-rrnBp (Zaslaver et al., 2006). *tetR* and *cmR* derived from pdCas9 (Addgene #44249) (Qi et al., 2013).


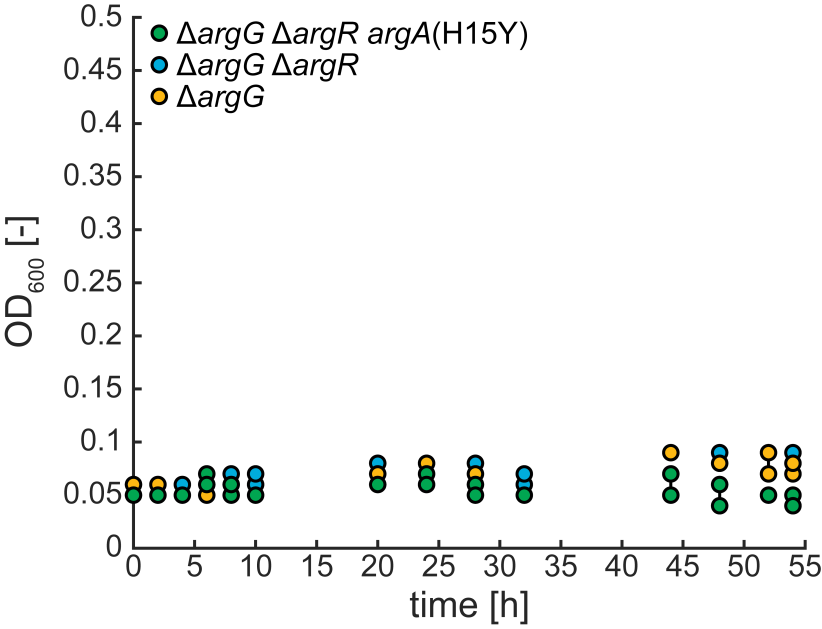


Suppl. Fig. 2. Growth of *argG* deletion strains during arginine starvation. Cells were first grown in minimal medium supplemented with arginine. Then, arginine was removed by washing, and cells were resuspended in minimal medium without arginine. The graph depicts the time course of the OD after the washing.

*
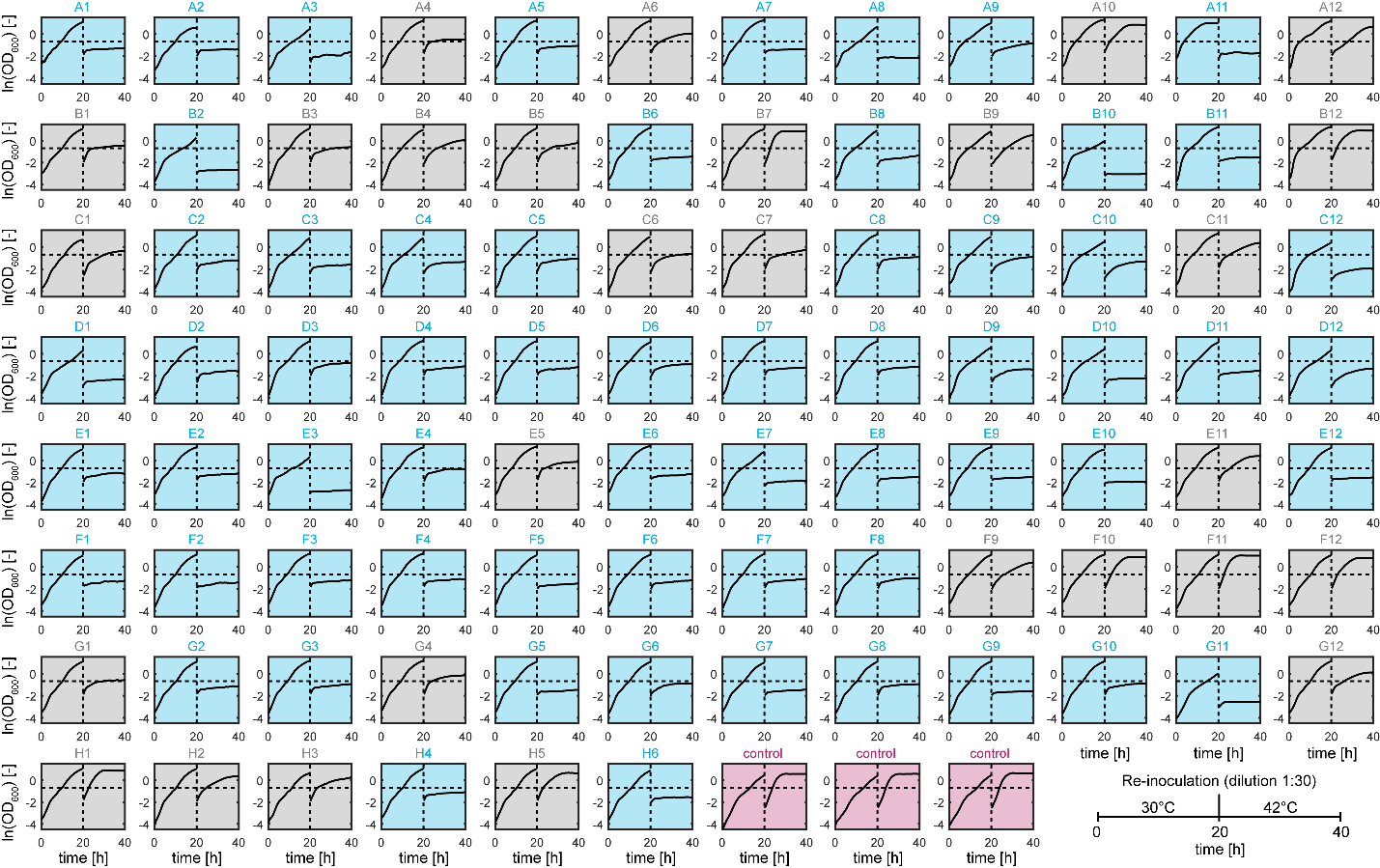
*

Suppl. Fig. 3. Growth of 90 randomly selected strains from the enriched *argG* library. Strains were cultivated in minimal medium for 20 h at 30°C, re-diluted by a factor of 30, and further incubated for 20 h at 42°C. 62 strains (blue) did not reach an OD of 0.5 after cultivation at 42°C. A control strain with wild-type ArgG is indicated in red.

**
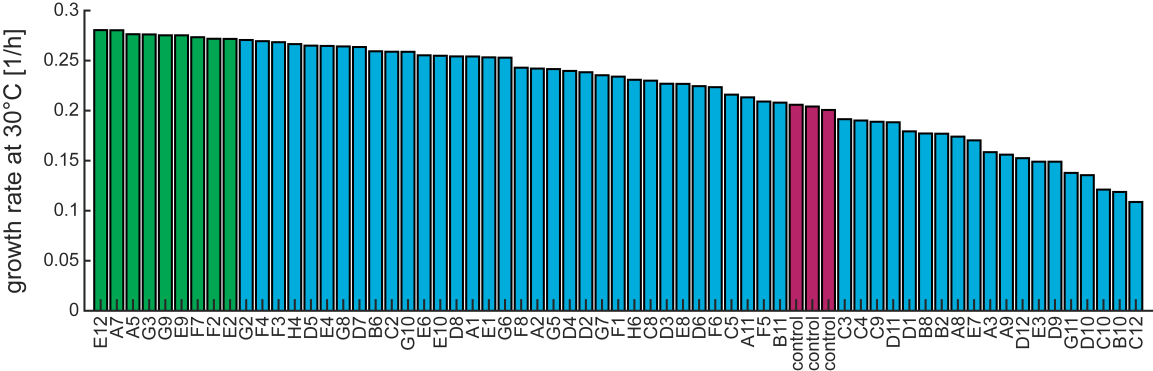
**

**Suppl. Fig. 4.** Growth rates at 30°C of 90 randomly selected strains from the enriched *argG* library. The nine fastest growing strains (green bars) were sequenced. A control strain with wild-type ArgG is indicated with red bars.


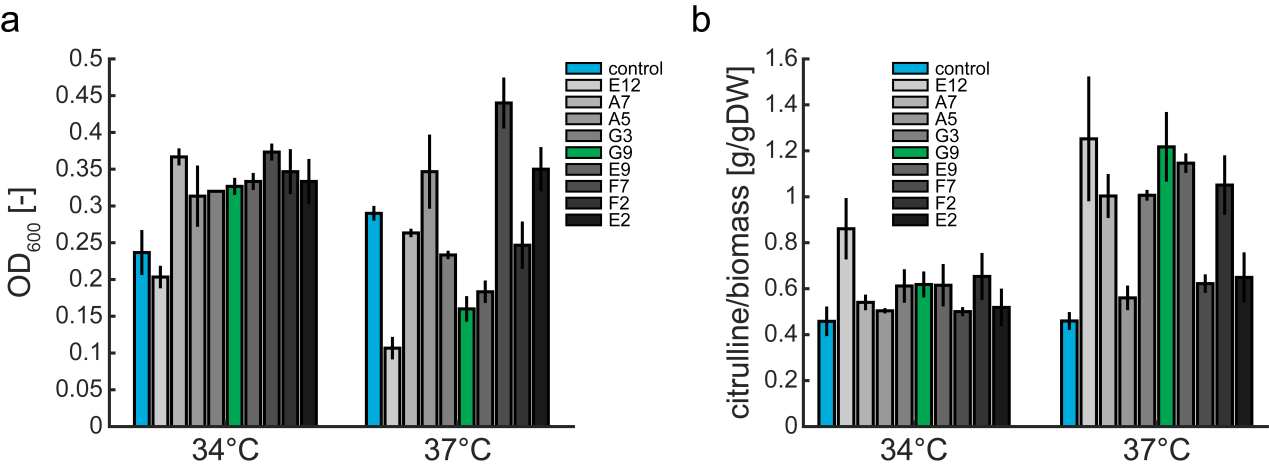


Suppl. Fig. 5. Growth and citrulline production of the doubly dysregulated citrulline producer in combination with the nine ArgG variants at intermediate temperatures. (a) Shown is the OD after 7 h of cultivation in minimal medium at 34°C and 37°C. Error bars show the standard deviation of n=3 cultures. (b) Biomass specific citrulline concentrations after 7 h of cultivation in minimal medium at 34°C and 37°C. Same strains as in (a). Error bars show the standard deviation of n=3 cultures.


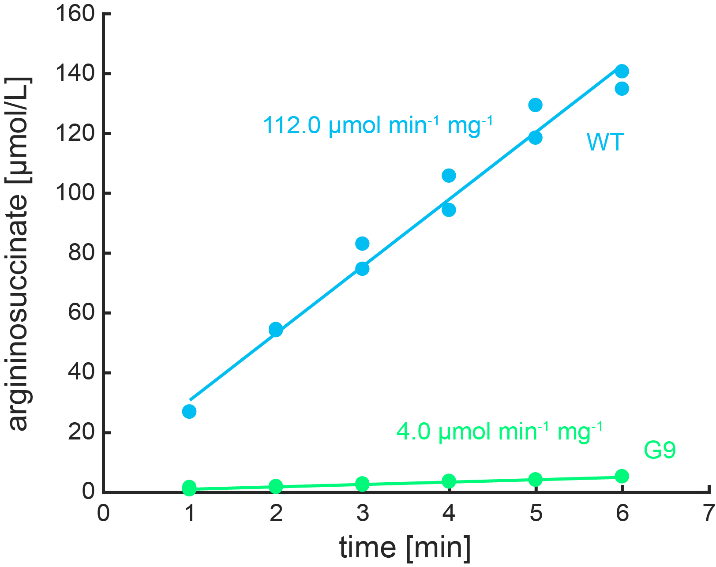


Suppl. Fig. 6. Specific enzyme activity of *E. coli* wildtype ArgG (WT) and ArgG variant G9 at 42°C. Enzymes were incubated for 1 h at 42°C before starting the reaction.


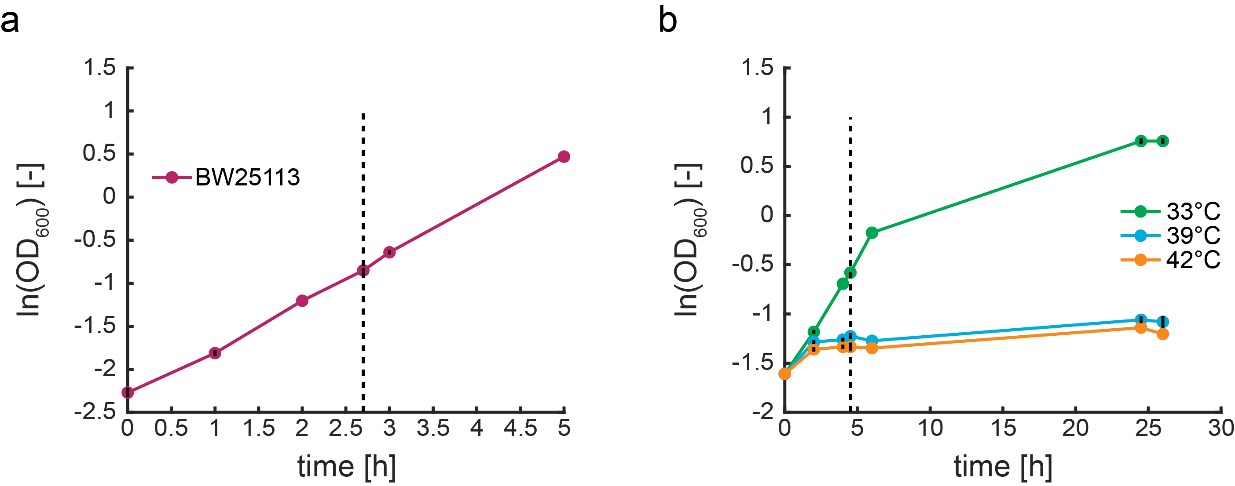


Suppl. Fig. 7. Growth of (a) wildtype *E. coli* and (b) the doubly dysregulated citrulline overproducer strain with the ArgG variant G9. Dotted lines indicate the time, at which samples for proteomics were taken. Dots are means, and error bars show the standard deviation of n=3 cultures.

**Supplementary Tables**

Suppl. Table 1. Strains, plasmids, and oligonucleotides used in this study.

| **Strains** | **Note** | **Reference/source** | |
| --- | --- | --- | --- |
| *Escherichia coli* MegaX DH10B T1R | Cat#C640003, used during cloning | Invitrogen, Thermo Fischer Scientific | |
| *E. coli* DH5α | Cat#18265017, used during cloning | Invitrogen, Thermo Fischer Scientific | |
| *E. coli* BL21(DE3) | Cat#C600003, used for protein expression | Invitrogen, Thermo Fischer Scientific | |
| *E. coli* BW25113 | parent strain of KEIO collection | (Datsenko and Wanner, 2000) | |
| *E. coli* JW3140 | Δ*argG*  (also BW25113 Δ*argG*) | (Baba et al., 2006) | |
| *E. coli* JW3140 (-Kan) | *E. coli* JW3140 with cured kanamycin resistance | This study | |
| *E. coli* JW3206 | Δ*argR* | (Baba et al., 2006) | |
| *E. coli* JW3140 Δ*argR* | Δ*argG*Δ*argR* | This study | |
| *E. coli* JW3140 Δ*argR* *argA*(H15Y) | Δ*argG*Δ*argR argA*^H15Y^ | This study | |
| *E. coli* JW3140//pBR322_TIMER | Used for transformation with the *argG* plasmid library | This study | |
| *E. coli* JW3140//pBR322_TIMER//pTS036(mutant) | non-screened *argG* library generated by error-prone PCR | This study | |
| *E. coli* JW3140 Δ*argR* *argA*(H15Y)//pBR322_TIMER//pTS036-*argG* | control in screening experiment | This study | |
| *E. coli* JW3140 Δ*argR* *argA*(H15Y)//pBR322_TIMER//pTS036#[variant] | variants as mentioned in the text | This study | |
| *E. coli* JW3140 Δ*argR* *argA*(H15Y)//pTS036-*argG* | dysregulated ctrulline overproduction strain with wild-type *argG* | This study | |
| *E. coli* JW3140 Δ*argR* *argA*(H15Y)//pTS036-G9 | dysregulated ctrulline overproduction strain with *argG* variant G9 | This study | |
| *E. coli* BL21(DE3)//pTS049 | overexpression of wildtype ArgG | This study | |
| *E. coli* BL21(DE3)//pTS050 | overexpression of ArgG variant G9 | This study | |
| **Plasmids** | **Note** | **Reference/source** | |
| pBR322_TIMER | single cell growth reporter | (Claudi et al., 2014) | |
| pCP20 | curation of kanamycin resistance from KEIO strain | (Datsenko and Wanner, 2000) | |
| pKDsgRNA-argA(H15Y) | sgRNA targeting *argA* near H15 | (Sander et al., 2019) | |
| pKDsgRNA-p15 | curation of the *Cas9* plasmid | Addgene plasmid #62656 (Reisch and Prather, 2015) | |
| pCas9-CR4 | *Cas9* plasmid for noSCAR | Addgene plasmid #62655 (Reisch and Prather, 2015) | |
| pdCas9 | template for cloning only | Addgene plasmid #44249 (Qi et al., 2013) | |
| pUA66-rrnBp | template for cloning only | (Zaslaver et al., 2006) | |
| pCA24N-argR | template for cloning only | (Kitagawa et al., 2006) | |
| pTS036-*argG* | pSC101, *cmR*, *tetR* under control of *proB*-promoter, wild-type *E. coli* *argG* under control of *pLtetO* | This study | |
| pTS036-*argG*(mutant) | plasmid library with mutagenized *argG* | This study | |
| pTS036#[variant] | variants as mentioned in the text | This study | |
| pTS036-G9 |  | This study | |
| pTS049 | pCA24N derivative with *E. coli* wild-type ArgG | This study | |
| pTS050 | pCA24N derivative with ArgG variant G9 | This study | |
| **Oligonucleotides** | | **Identifier** | **Use** |
| ATGACGACGATTCTCAAGCATCTC | | argG-amp-F | pTS036-*argG*, pTS036-G9 |
| TTACTGGCCTTTGTTTTCCAGATTC | | argG-amp-R | pTS036-*argG,* pTS049 |
| AATCTGGAAAACAAAGGCCAGTAAATGTGGATCCCCAGACCTGCAGGCATG | | argG-pSC101-F | pTS036-*argG* |
| GAGATGCTTGAGAATCGTCGTCATAGATCCTTTCTCCTCTTTAGATCTTTTGAATTCTTGGTC | | argG-pSC101-R | pTS036-*argG* |
| TTACTGGCCTTTGTTTTCCAGACTC | | G9-ampR | pTS036-G9, pTS050 |
| GCCTTCTCCTGCTCTCCCTTAAGCGC | | argG-F | sequencing |
| GAGACTCACGGGTTGTGGATGCAAACCATG | | argG-R | sequencing |
| GATAAACTAAGATATGTTGCTCCGCTGCCG | | argR-F | sequencing |
| GTATTCATTGTGTGAATGACATGTCGCAG | | argR-R | sequencing |
| GTGGTAAAGGAACGTAAAACCGAGTTG | | argA-F | sequencing |
| TTACCCTAAATCCGCCATCAACAC | | argA-R | sequencing |
| GTGGTAAAGGAACGTAAAACCGAGTTGGTCGAGGGATTCCGCTATTCCGTTCCCTATATCAATACCCACCGGGGAA | | argA_H15Y_rec2 | noSCAR *argA*(H15Y) (Sander et al., 2019) |
| ACGACGATTCTCAAGCATCTCCCG | | argG-noATG-F | pTS049, pTS050 |
| TCTGGAAAACAAAGGCCAGTAAGGCCTATGCGGCCGCTAAGGGTCGACCTG | | ASKA-His-ArgG-F | pTS049, pTS050 |
| GAGATGCTTGAGAATCGTCGTGGCCCTCAGGGCCGGATCCGTATGGTG | | ASKA-His-ArgG-R | pTS049, pTS050 |

Suppl. Table 2. Mutations and predicted ΔΔ*G* values of 9 ArgG variants.

| **ArgG variant** | **Non-** **synonymous mutations** | **No. of synonymous mutations** | **ΔΔ*G****  **[kcal mol^-1^]** |
| --- | --- | --- | --- |
| E12 | Y39H, R59S, Q152H, V223A, K252E, P384L | 3 | 6.1 |
| A7 | L29Q, Q86L, I163F, I356N | 1 | 4.4 |
| A5 | E62D, I356F | 0 | 8.8 |
| G3 | F100Y, D164V, E235D, G269S, I311F, S339P | 1 | 9.0 |
| G9 | L29P, E228V, R416H, Q438R, N441S | 6 | 6.0 |
| E9 | E66D, L308M, N368D | 1 | 1.1 |
| F7 | G272S, T375S | 2 | 7.5 |
| F2 | A36V, L210H, M260V, F337L | 1 | 9.9 |
| E2 | K34R, N92D, M197V, S229C, E235D, F420Y | 2 | 5.3 |

*****ΔΔ*G* = Δ*G*^fold^_mutant_ - Δ*G*^fold^_wildtype_, estimated with FoldX 5.0 (Guerois et al., 2002; Schymkowitz et al., 2005) using a re-refined ArgG crystal structure (1k92 from PDB_REDO) (Joosten et al., 2011; Lemke and Howell, 2001)**.**

Suppl. Table 3. Citrulline concentrations, biomass-specific citrulline yields, and citrulline production rates in different experiments.

|  | **Doubly dysregulated ArgG deletion strain (Fig. 3) (n=2)** | **G9-based strain at 42°C (Fig. 4) (n=3)** | **G9-based strain in bioreactor (Fig. 5) (n=2)** |
| --- | --- | --- | --- |
| citrulline concentrations [g L^-1^] | 0.024 ± 0.003 (after 10 h) | 0.033 ± 0.002 (after 7 h) | 3.09 ± 0.23 (after 45 h) |
| yields [g g_DW_^-1^] | 1.32 ± 0.18 (after 10 h) | 1.35 ± 0.14 (after 7 h) | 5.48 ± 0.84 (after 45 h) |
| production rates [mmol g_DW_^-1^ h^-1^] | 0.73 ± 0.07 (first 10 h) | 1.10 ± 0.12 | 1.01 ± 0.13  (only production phase) |

**Supplementary Methods**

*S1. Sample preparation for proteomics*

Samples for proteomics were prepared by transferring 10^9^ cells to a 15 mL reaction tube and centrifugation (4°C, 4000 rpm, 10 min). The supernatant was removed, and the cell pellet resuspended in 10 mL 4°C-cold phosphate buffered saline (PBS, 8 g/L NaCl, 0.2 g/L KCL, 0.2 g/L KH_2_PO_4_, 1.15 g/L Na_2_HPO_4_, pH 7.4). After centrifugation (4°C, 4000 rpm, 10 min), the cell pellet was resuspended in 1 mL 4°C-cold PBS and transferred to 2 mL reaction tubes. Cells were pelletized by centrifugation (4°C, 17.000 g, 5 min), the supernatant removed, and the pellet stored at -80°C. 300 µL lysis buffer (2% Na-lauroyl sarcosinate in 100 mM NH_4_HCO_3_) were added to the cell pellet, and incubated at 90°C for 16 min under shaking at 1200 rpm. Remaining cell pellet was resuspended by 25 s of sonication. Samples were centrifuged (15.000 rpm, 20°C, 5 min). The protein concentration in the supernatant was determined with a BCA Protein Assay Kit (Thermo Fisher, #23252). 7.5 µL of 5 mM tris(2-carboxyethyl)phosphine (TCEP) were added to the samples and incubated at 90°C for 10 min under shaking at 1200 rpm. After cooling of the samples, 10 µL of 10 mM iodacetamide were added and incubated at 25°C for 30 min under shaking of 500 rpm. 50 µg of protein was transferred to new reaction tubes. 10 µL of 0.1 g/L trypsin were added and incubated overnight at 30°C under shaking of 750 rpm. 90 µL of 5% trifluoroacetic acid and 50 µL of 10% HCl were added and incubated for 10 min at room temperature. Samples were centrifuged for (10 min, 15.000 rpm, 4°C). The supernatant was transferred to CHROMABOND Spincolumns (Macherey-Nagel) that were conditioned with 500 µL of acetonitrile and equilibrated with 500 µL and 150 µL 0.1% TFA. After loading, the peptides were washed with 500 µL 0.1% TFA in 5:95 acetonitrile:water and eluted with 400 µL 0.1% TFA in 50:50 acetonitrile:water. Peptides were concentrated and dried under vacuum at 50°C. Peptides were dissolved in 100 µL 0.1% TFA by 25 s of sonication and incubation at 22°C under shaking at 1200 rpm for 5 min.

**References**

Baba, T., Ara, T., Hasegawa, M., Takai, Y., Okumura, Y., Baba, M., Datsenko, K.A., Tomita, M., Wanner, B.L., Mori, H., 2006. Construction of *Escherichia coli* K‐12 in‐frame, single‐gene knockout mutants: the Keio collection. Mol. Syst. Biol. 2, 1. https://doi.org/10.1038/msb4100050

Claudi, B., Spröte, P., Chirkova, A., Personnic, N., Zankl, J., Schürmann, N., Schmidt, A., Bumann, D., 2014. Phenotypic Variation of Salmonella in Host Tissues Delays Eradication by Antimicrobial Chemotherapy. Cell 158, 722–733. https://doi.org/10.1016/j.cell.2014.06.045

Datsenko, K.A., Wanner, B.L., 2000. One-step inactivation of chromosomal genes in *Escherichia coli* K-12 using PCR products. PNAS 97, 6640–6645. https://doi.org/10.1073/pnas.120163297

Davis, J.H., Rubin, A.J., Sauer, R.T., 2011. Design, construction and characterization of a set of insulated bacterial promoters. Nucleic Acids Res. 39, 1131-1141. https://doi.org/10.1093/nar/gkq810

Elowitz, M.B., Leibler, S., 2000. A synthetic oscillatory network of transcriptional regulators. Nature 403, 335-338. https://doi.org/10.1038/35002125

Guerois, R., Nielsen, J.E., Serrano, L., 2002. Predicting Changes in the Stability of Proteins and Protein Complexes: A Study of More Than 1000 Mutations. J. of Mol. Biol. 320, 369–387. https://doi.org/10.1016/S0022-2836(02)00442-4

Joosten, R.P., te Beek, T.A.H., Krieger, E., Hekkelman, M.L., Hooft, R.W.W., Schneider, R., Sander, C., Vriend, G., 2011. A series of PDB related databases for everyday needs. Nucleic Acids Res. 39, D411–D419. https://doi.org/10.1093/nar/gkq1105

Kitagawa, M., Ara, T., Arifuzzaman, M., Ioka-Nakamichi, T., Inamoto, E., Toyonaga, H., Mori, H., 2006. Complete set of ORF clones of *Escherichia coli* ASKA library (A Complete Set of *E. coli* K-12 ORF Archive): Unique Resources for Biological Research. DNA Res. 12, 291–299. https://doi.org/10.1093/dnares/dsi012

Lemke, C.T., Howell, P.L., 2001. The 1.6 Å Crystal Structure of *E. coli* Argininosuccinate Synthetase Suggests a Conformational Change during Catalysis. Structure 9, 1153–1164. https://doi.org/10.1016/S0969-2126(01)00683-9

Lutz, R., Bujard, H., 1997. Independent and Tight Regulation of Transcriptional Units in *Escherichia coli* via the LacR/O, the TetR/O and AraC/I_1_-I_2_ Regulatory Elements. Nucleic Acids Res. 25, 1203-1210. https://doi.org/10.1093/nar/25.6.1203

Qi, L.S., Larson, M.H., Gilbert, L.A., Doudna, J.A., Weissman, J.S., Arkin, A.P., Lim, W.A., 2013. Repurposing CRISPR as an RNA-Guided Platform for Sequence-Specific Control of Gene Expression. Cell 152, 1173–1183. https://doi.org/10.1016/j.cell.2013.02.022

Reisch, C.R., Prather, K.L.J., 2015. The no-SCAR (Scarless Cas9 Assisted Recombineering) system for genome editing in *Escherichia coli.* Sci. Rep. 5, 15096. https://doi.org/10.1038/srep15096

Sander, T., Farke, N., Diehl, C., Kuntz, M., Glatter, T., Link, H., 2019. Allosteric Feedback Inhibition Enables Robust Amino Acid Biosynthesis in *E. coli* by Enforcing Enzyme Overabundance. Cell Syst. 8, 66-75.e8. https://doi.org/10.1016/j.cels.2018.12.005

Schymkowitz, J., Borg, J., Stricher, F., Nys, R., Rousseau, F., Serrano, L., 2005. The FoldX web server: an online force field. Nucleic Acids Res. 33, W382–W388. https://doi.org/10.1093/nar/gki387

Zaslaver, A., Bren, A., Ronen, M., Itzkovitz, S., Kikoin, I., Shavit, S., Liebermeister, W., Surette, M.G., Alon, U., 2006. A comprehensive library of fluorescent transcriptional reporters for *Escherichia coli*. Nat. Methods 3, 623–628. https://doi.org/10.1038/nmeth895
